# Proteomic Biomarkers in VTE: From Discovery to Clinical Translation

**DOI:** 10.3390/jcm15103745

**Published:** 2026-05-13

**Authors:** Tengyi Cai, Prahlad Ho, Julie Wang

**Affiliations:** 1 Northern Clinical Diagnostics & Thrombovascular Research (NECTAR), Northern Health, Melbourne, VIC 3076, Australia; tengyi.cai@nh.org.au (T.C.); prahlad.ho@nh.org.au (P.H.); 2 Northern Pathology Victoria, Melbourne, VIC 3076, Australia; 3 Department of Haematology, Northern Health, Melbourne, VIC 3076, Australia; 4 Department of Medicine, Northern Health, University of Melbourne, Epping, VIC 3076, Australia; 5 Australian Centre for Blood Diseases, Monash University, Melbourne, VIC 3004, Australia; 6 School of Health and Biomedical Sciences, RMIT University, Bundoora, VIC 3083, Australia

**Keywords:** VTE, proteomics, plasma, coagulation, thromboinflammation

## Abstract

**Background**: Venous thromboembolism (VTE) remains a leading cause of global morbidity and mortality. The pathophysiological mechanisms leading to VTE are still not fully elucidated. Diagnostic biomarkers for VTE lack specificity, and biomarkers to guide the duration of anticoagulation therapy have not yet entered routine clinical practice. Proteomics has emerged as a high-throughput approach for novel biomarker discovery, offering insights into the complex biological processes across the VTE continuum. This review explores current VTE proteomic research and discusses the challenges and gaps hindering clinical translation. **Methods**: We performed a narrative review based on a search of Scopus and PubMed for original human proteomic studies published between 1995 and July 2025. Results were limited to whole-blood, plasma or serum-based studies. **Results**: A total of 1190 studies were retrieved, of which 27 studies were included in this review. Studies were mainly plasma-based. Studies compared VTE patients to non-VTE controls, different VTE subtypes, and various provoked VTE cohorts. Broad themes identified proteomic signatures involving dysregulated coagulation, complement activation, inflammation, and platelet activation. **Conclusions**: Current proteomic evidence supports VTE as a systemic immunothrombotic disorder that shows key differences even years before developing VTE. Proteomic research in VTE holds the promise to identify biomarkers that may aid in the diagnosis and guide management of VTE. However, most proteomic findings remain exploratory to date and methodologies are varied across studies. Future studies should prioritise the workflow standardisation and validate promising biomarker panels in large-scale, prospective, longitudinal cohorts.

## 1. Introduction

Venous thromboembolism (VTE), including deep vein thrombosis (DVT) and pulmonary embolism (PE), is one of the leading causes of morbidity and mortality globally, affecting over 10 million people annually and causing over 500,000 deaths [1,2]. Recent epidemiological studies have reported that VTE hospitalisations increased from about 110 to 178 per 100,000 population between 1998 and 2022, driven largely by increasing PE hospitalisations (from 40 to 122 per 100,000 population), whereas DVT hospitalisations decreased from 69 to 56 per 100,000 over the same period [3]. Despite its prevalence and impact, the exact pathophysiological mechanism leading to VTE remains unclear. Current knowledge suggests an interplay of environmental factors and modifiable lifestyle factors in susceptible individuals, but the biological pathways mediating these effects are not fully understood [4]. Additionally, VTE is a chronic, relapsing condition with recurrence rates after a first episode of VTE being 5–7% per year and reaching 36% within 10 years after unprovoked VTE [5]. The risk factors for recurrent VTE include previous unprovoked VTE, obesity, male sex, autoimmune conditions including inflammatory bowel disease yet accurately identifying individuals within this group remains challenging. Existing clinical scores lack precision and rely largely on clinical factors [1,6]. Therefore, novel, specific biomarkers are urgently needed to aid diagnosis and identify individuals at high risk of de novo and recurrent VTE, improving clinical decision-making and patient outcomes.

Proteomics has emerged as a powerful and increasingly accessible approach for novel biomarker discovery. Over the last decade, significant technological advancements in mass spectrometry (MS) and affinity-based assays have enabled large-scale, high-throughput, and comprehensive measurement of the proteome in a small volume of blood or tissue [7]. By capturing not only protein abundance but also post-translational modifications and pathway-level changes, proteomic profiling may characterise the complex biological processes leading to VTE. In this narrative review, we summarise current proteomic studies in VTE and specifically focus on the predictive potential of these findings to transform diagnosis and improve patient outcomes.

## 2. Materials and Methods

### 2.1. Search Strategy and Selection Criteria

We searched Scopus and PubMed-indexed online databases for studies published from January 1995 to July 2025. The specific search terms used for PubMed database search are detailed in Appendix A. Briefly, studies included in this narrative review were identified using the search terms [‘venous thromboembolism’ OR ‘venous thrombosis’ OR ‘deep vein thrombosis’ OR ‘pulmonary embolism’ OR ‘VTE’ OR ‘DVT’ OR ‘PE’] AND [‘proteomics’ OR ‘plasma/serum/circulating protein’ OR ‘mass spectrometry’ OR ‘LC-MS’ OR ‘tandem mass spectrometry’ OR ‘shotgun proteomics’ OR ‘protein profiling’], as well as derivatives of these terms. Searches included all forms of VTE and all proteomics-related analytical approaches. Studies were limited to original research articles involving human subjects, published in the English language between 1995 and 2025. Studies retrieved using these search parameters were screened by the author (TC) focusing on titles and abstracts for eligibility based on the inclusion and exclusion criteria. Full texts of the chosen articles were assessed by TC to confirm this.

### 2.2. Inclusion and Exclusion Criteria

Inclusion criteria: (I) Study investigates DVT, PE, or VTE; (II) proteomic analysis or protein profiling performed; (III) human-derived whole-blood, plasma, or serum samples used; (IV) original research article; (V) published in English; and (VI) publication date between 1995 and 2025.

Exclusion criteria: (I) Animal-only studies; (II) review articles, meta-analyses, or conference abstracts; (III) studies unrelated to proteomic techniques or without protein-level data; (IV) studies that utilised samples other than whole-blood, plasma, or serum; (V) cancer-related VTE studies; and (VI) articles not available in full text in English.

### 2.3. Data Extraction and Quality Assessment

Included studies were reviewed by TC and JW, with disagreement resolved by consensus. Data extracted included study design, patient group and size, sample collection time point and sample type, analysis technique/s, protein identification findings and relative clinical significance of candidate proteins from each study.

## 3. Results

### 3.1. Study Overview

**Figure 1:**
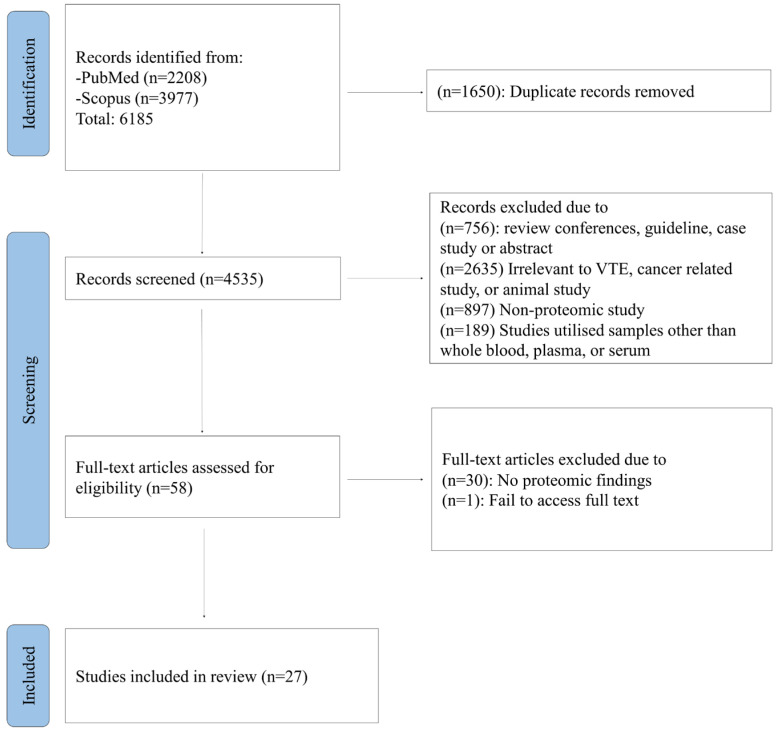
Summary of the study selection process for the review.

Figure 1 shows the flow diagram for study inclusion and exclusion. In total, 4535 unique studies were identified using our systematic search strategy. Papers were initially screened by their titles and abstracts, with most studies (n = 4477) excluded for not fulfilling the inclusion parameters. The 58 remaining studies were reviewed in full. After excluding 30 studies due to no protein being identified and one study due to the full text being unavailable [8], 27 studies were finally included in this review. The full name of the proteins are summarised in the Abbreviations list.

### 3.2. Studies Comparing VTE Patients and Non-VTE Controls

To date, 16 studies (Table 1) have investigated proteomic differences between patients with VTE and non-VTE controls [9,10,11,12,13,14,15,16,17,18,19,20,21,22,23,24]. The sample collection time with respect to VTE varied significantly across studies, ranging from up to 12.6 years before the VTE event [17] to 4 years following [9]. In more detail, five studies collected samples before the VTE event [11,17,19,21,22,23], seven studies shortly after the VTE event (at diagnosis or within 2 weeks following diagnosis) [12,14,15,16,18,20,24], and two studies with sample collected after a longer period following the VTE event [9,10].

#### 3.2.1. Pre-VTE Studies

The six studies [11,17,19,21,22,23] with samples collected before the VTE event harnessed samples from large biobanks, including the Tromsø study [11,17], Swedish cohort [23], UK biobank [19,22] and deCODE Icelanders cohort [21]. Findings of these studies revealed consistent proteomic profiles in patients who later developed VTE, characterised by dysregulated coagulation, platelet activation, complement and inflammation.

Identified proteomic profiles were most similar between the large studies using GWAS data originating from the UK biobank [19,22] and Icelander biobanks [21], likely due to their similar methodologies (Proteome-wide Mendelian Randomization (MR)), similar cohorts (European), and identical proteomics platform used (SomaScan). These studies integrated genetic GWAS data with proteomic PWAS data and identified a core set of proteins that were associated with increased VTE risk, including coagulation Factors II, V, XI, and protein C, platelet activation/adhesion proteins (PLCG2, GP6, PLEK), inflammatory mediators (KNG1, KLKB1, IL-6R, ANGPT1, ULBP2, IL18BP, CCL25, VSIG2), and other proteins involved in vascular remodelling (SERPINA1, SERPINE2, SERPINA10, THBS2, EFEMP1, LRP4, ICAM2, MAN1A2). Pathway analysis based on these differently expressed proteins confirmed enrichment of complement and coagulation cascades, platelet–endothelial activation, immune response, and inflammation. Complementing these findings, Yuan et al. [23] used a Swedish prospective cohort with targeted Olink proteomics followed by MR, indicating the causal associations of vWF, PAI-1, EPHB4, TYRO3, TNFRSF11A, BOC and CHI3L1 with VTE risk.

The smaller study by Jensen et al. [11] focused on individuals that developed the first lifetime VTE from the Tromsø cohort, and identified 46 candidate plasma proteins. In this study, TTR, ProZ and DJ-1 emerged as the strongest predictors of VTE, alongside other candidate proteins including Factor IX, Gal-3, S100A8/A9 and protein Z-dependent protease inhibitor. These proteins were involved in regulation of Factor X, oxidative stress, neutrophil activation and complement, also pointing toward an activated coagulation–inflammation axis among patients even years before the VTE event. Interestingly, when the authors re-analysed the initial untargeted proteomic dataset with a more refined statistical approach in 2022 [17], only LBP was identified as a new and potential biomarker of future DVT. This underscores the critical importance of rigorous data analysis in biomarker discovery. In this study, LBP was further validated by ELISA in a larger, nested case–control validation cohort consisting of 410 VTE cases and 834 controls, which further confirmed that higher baseline LBP predicted future DVT in women. The authors postulated that the elevation of LBP indicated low-grade endotoxemia and chronic inflammation, which could activate endothelial cells and monocytes and lead to thrombosis. LBP is an acute-phase protein produced primarily in the liver, which is a critical mediator in immune response in bacterial infection and involved in reversing the amyloidosis of fibrin in type 2 diabetes patients [25,26]. LBP has also been identified in the study by Bryk et al. [27], which demonstrated fibrin clots prepared from plasma of acute PE patients contained 144% higher levels of LBP compared to clots of healthy controls. However, further clinical studies are required to confirm the utility of this promising biomarker in VTE.

Despite similarities, there are also notable differences between the reported proteomic findings, which may be partially attributed to study design and analytical framework. The Mendelian randomisation based studies [19,21] investigated lifelong effects of genetically proxied protein levels on VTE risk in multiple and large case–control datasets, capturing “causal” proteins that shape baseline susceptibility. In contrast, the studies by Jensen et al. [11] directly determined protein abundances in stored plasma samples by untargeted MS-based proteomics from a relatively smaller VTE patient cohort, so results can also be affected by sampling timepoint, underlying inflammation, and sample handling/storage.

Overall, the four pre-VTE studies showed that individuals who later develop VTE already presented reproducible proteomic profiles years before developing VTE. The proteomic changes encompassed coagulation, platelet activation, complement and inflammation. These studies primarily use proteomic profiling as a discovery approach to nominate candidate proteins for future VTE risk prediction and are important to advance our understanding of the underlying mechanisms leading to VTE in susceptible individuals. However, protein candidates from these studies still require further independent replication, correlation with coagulation factor function and clinical validation, particularly if these proteins independently predict future VTE risk.

#### 3.2.2. Post-VTE Studies: Acute Phase (Within 2 Weeks)

Seven proteomic studies [12,14,15,16,18,20,24] collected samples within 2 weeks of acute VTE presentation, and the majority of these were from the time of presentation, or within 24 h of VTE diagnosis. Similar to the pre-VTE studies, acute VTE phase plasma proteomics consistently reported changes in coagulation proteins (ATIII, P-selectin, VWF, TFPI, Fibrinogen, ST2), platelet proteins (CO1A1, CO3A1) and inflammatory markers (OPN, HP, SAA1, S100A8, LRG1, Gal3BP, GSN) [12,14,15,16,28].

Among the many proteins nominated by discovery proteomic studies, CFHR5 (complement factor H-related 5) merits particular attention because it has progressed beyond initial identification toward validation and mechanistic follow-up. Iglesias et al. [18] identified CFHR5 as a novel plasma biomarker associated with acute VTE across multiple independent VTE European patient cohorts and showed that this signal remained reproducible throughout the course of VTE treatment. CFHR5 competes with complement factor H in binding C3b, resulting in deregulation of the alternative complement pathway and enhancement of C3b activity. The authors showed through several cohorts that CFHR5 was associated with VTE independent of D-dimer and CRP, enhanced VTE diagnosis accuracy, and a trend towards VTE recurrence in individuals with an initial unprovoked VTE. This was supported mechanistically by their functional testing, showing higher CFHR5 correlated with increased thrombin generation and platelet activation. However, Mendelian Randomization of CFHR5 based on GWAS meta-analysis across FARIVE, RETROVE, and MARTHA cohorts did not support a direct causal effect of CFHR5 on VTE, and the authors postulate that this is due to multiple biologic players interacting with the CFHR5 locus and with pleiotropic effects themselves.

Nevertheless, the afore-mentioned study adds to the body of evidence linking complement activation or dysregulation to VTE. Other plasma studies in acute VTE have similarly reported complement-driven signatures [15,16,24]. Han et al. [15] and Granholm et al. [16] found increased C9, CFH and α2-glycoprotein in acute PE, supporting alternative pathway activation during VTE. Coagulation and inflammatory pathways were also activated. These plasma-based findings are further reinforced by Bryk et al. [27], who analysed the proteomic profile of fibrin clots generated ex vivo from patient plasma. Their discovery of increased complement components C5–C9 within the clot structure mirrors the systemic inflammatory and complement-driven response observed in direct plasma studies.

Wang et al. [20] identified coagulation and platelet-related proteins (PDGFB, VWF, FIX) together with inflammatory and growth factor proteins (Gal-3, CHI3L1, IL-1), with pathway analyses enriched for inflammation, coagulation and cytokine–cytokine receptor interaction. Additionally, Pallares Robles et al. [29] provided another angle by considering FXI activity as a clinically relevant reference point to define broader proteomic signatures in VTE. In this study, FXI activity was found higher during the acute VTE event than at 12 months, with 21 FXI-associated proteins identified in acute VTE, 66 at 12-month follow-up, and seven proteins shared across both timepoints (FXI:Ag, REG3A, PIGR, IL7R, LTBP2, PRDX1 and GALNT3). These shared and time-specific proteins are also broadly consistent with Wang et al. [20], but also to thromboinflammatory pathways involving IL-1, TNFR2, NF-κB and TLR4 signalling, together with apoptosis, extracellular matrix interaction and lipid metabolism.

Findings across these studies also suggest that acute VTE triggers a complex dysregulation of the fibrinolytic system. Granholm et al. [16] observed decreased TAFI in acute VTE patients, which likely reflects the systemic consumption as it binds to and stabilises the fibrin matrix, protecting the clot from premature degradation. While Bryk-Wiązania et al. [30] revealed that purified plasminogen from acute PE patients on admission undergoes complex post-translational modifications, including significant oxidative, carbonyl, and S-nitrosyl modifications, as well as reduced 8-isoprostane levels. These changes resulted in a 12.8% decrease in clot lysis potential, indicating that oxidative stress directly impairs the efficiency of plasminogen. This stabilisation provides the necessary environment for TNC and LRG1 to serve as a matricellular signal for tissue repair and angiogenesis [15,16].

Overall, proteomic profiling of acute VTE reveals a biological landscape dominated by complement activation, coagulation, and inflammatory signalling as well as dysregulated fibrinolysis. Additionally, matricellular proteins like TNC and LRG1 are upregulated, reflecting active tissue repair and angiogenesis. These studies, conducted at VTE presentation or within 2 weeks of diagnosis, may identify potential biomarkers for diagnosis. Further clinical validation is needed to investigate these promising findings.

#### 3.2.3. Post-VTE Studies: Post-Acute

Only two studies used samples from after the VTE episode (up to 4 years post) [9,10]. Flores-Nascimento et al. [9] reported higher levels of complement C4A, inter-α-trypsin inhibitors and SAA, with lower APOA4 and AHSG in DVT patients, indicating persistent complement activity, acute-phase response and altered lipid transport long after the acute DVT event. By contrast, Bruzelius et al. [10] identified coagulation and platelet-related proteins (PDGFB, VWF) together with inflammatory protein, HIEVP1, and scavenger of reactive oxygen species, GPX3, which indicated the potential post-VTE inflammation and subsequent extracellular matrix turnover. Overall, these pro-inflammatory, pro-coagulant and pro-remodelling profiles could plausibly contribute to VTE recurrence and other post-thrombotic complications.

These post-VTE studies suggest a development toward a more chronic inflammation and remodelling-dominated phenotype. This convalescent stage is characterised by sustained activation of coagulation and platelet pathways, alongside persistent inflammatory signatures involving proteins such as SAA and growth factors like PDGFB. Complement activation appears less dominant, while ongoing extracellular matrix turnover and altered lipid metabolism suggest a prolonged vascular response. These studies support a specific proteomic pattern in high VTE risk patients. However, no studies to date have compared the proteomic profiles of patients with VTE recurrence to those without. Further studies are required to explore this clinically important question.

### 3.3. Comparing Proteomics of Isolated PE or DVT and High-Risk to Low-Risk PE

Six studies aimed to identify novel diagnostic biomarker to differentiate between VTE subtypes and were conducted mainly at the time of presentation. Specifically, three studies [31,32,33] compared patients with isolated PE, isolated DVT and combined DVT and PE; two studies [15,34] investigated proteomic differences between high-risk and intermediate/low-risk PE; and one study [20] compared patients with lower extremity DVT to Non-lower extremity DVT.

The exact mechanisms leading to thrombus embolisation from lower limb to the lungs resulting in PE are not understood. Proteomic studies show that patients with isolated DVT, isolated PE and PE and DVT display distinct differences, reflecting varying degrees of inflammation, endothelial activation, and platelet reactivity. In patients presenting with isolated PE, Ten Cate and Prochaska et al. [31] identified a unique five-protein signature (IFN-γ, GDNF, GALNT3, PADI2, IL-15Rα), reflecting upregulated inflammation, oxidative-stress pathways and markers of pulmonary surfactant disturbance. Razzaq et al. [33] utilised an artificial neural network to identify top proteomic predictors like VCL, VWF, ROCK1, COX4I2, and FABP6, which are involved in platelet adhesion, coagulation, and inflammation. Baidildinova et al. [32] discovered that extracellular vesicles and shedding release PE-specific platelet-related proteins that cluster in immunoreceptor signalling, chemotaxis and pathogen clearance pathways. In addition, machine learning models achieved high discriminatory power, with area under the operative curve (AUC) of 0.96 (isolated PE vs. isolated DVT) [31], and AUC of 0.79 for PE prediction from a VTE cohort [33]. For combined DVT+PE patients, proteomic profiles appear to be driven by pathways predominantly involved in platelet activation, grouped into tissue remodelling, and oxidative stress, while sharing inflammatory features with isolated PE. Unlike isolated PE, the platelet releasate in DVT+PE is driven predominantly by platelet α-granule secretion rather than surface shedding [31,32,33]. Models differentiating combined DVT+PE from isolated DVT demonstrated robust performance, with AUCs of 0.89 reported by both Ten Cate et al. [31] and Baidildinova et al. [32].

In contrast, patients with isolated DVT demonstrated proteomic evidence of local clot formation and matrix remodelling [33]. In vitro functional assays demonstrated platelets from isolated DVT patients possess a significantly higher capacity for aggregation and tissue factor-triggered thrombin generation and could indicate a more pro-coagulant platelet phenotype that may facilitate thrombus stabilisation [32]. However, these studies were sampled during acute phase of VTE, thus the proteomic signatures are more likely to capture downstream consequences and acute systemic response, which may not be able to distinguish different subtypes before VTE event.

Proteomic profiling may also reflect disease severity within patients presenting with PE. In the context of PE, two studies indicate that high-risk PE is molecularly distinct from low/intermediate-risk PE, characterised by stronger acute inflammatory response and more severe haemolysis. Insenser et al. [34] revealed that high-risk PE patients exhibit significantly reduced levels of haptoglobin and hemopexin, in agreement with earlier animal and human studies [34]. This pattern suggests that severe hemodynamic turbulence and shear stress across the cardiac valves and pulmonary tree in high-risk PE promotes haemolysis, and excess free haemoglobin rapidly consumes scavenging proteins, depleting their plasma levels and highlighting a pathophysiological mechanism not seen in low-risk PE. Han et al. [15] also identified 70 significant altered plasma proteins between high-risk and low/intermediate-risk PE, and the relative pathway analysis of these proteins showed enrichment in blood coagulation, acute inflammatory response, positive regulation of chemokine production, and receptor-mediated endocytosis. This study identified markers including acute-phase and immune markers like S100A, reflecting the intense neutrophil-mediated inflammation, while TNC upregulation signals the activation of vascular tissue repair mechanisms in response to severe injury.

Regarding the differentiation of DVT phenotypes, Wang et al. [20] utilised cytokine arrays to distinguish the differences between patients with lower extremity DVT and Non-lower extremity DVT. They found that the lower extremity DVT phenotype is characterised by a heightened inflammatory and immune signalling profile, with proteins such as Gal-3, IL-1, Factor IX and CHI3L1 being significantly higher in lower extremity DVT. This suggested that the formation of a deep vein thrombus is intrinsically linked to a specific upregulation of immune modulation and endothelial interaction pathways.

Proteomic profiling to date suggests VTE subtypes are biologically heterogeneous. Patients with isolated DVT and/or PE, as well as high- vs. lower-risk PE subtypes, can be differentiated with varying accuracy by examining protein patterns alone, which reflect varying degrees of inflammation, endothelial activation, and platelet involvement between subtypes. Machine learning models leveraging these differences may achieve high accuracy in distinguishing VTE subtypes, demonstrating the potential of proteomic biomarkers for improved diagnosis, and therapeutic options may differ based on different subtypes.

### 3.4. Different Provoked VTE Cohorts

VTE often occurs after transient provoking factors like surgery, trauma, immobility, flights, and hormonal therapy. However, the exact mechanism of why these factors provoke VTE is still unclear. Studying the proteomic profile of different provoked VTE cohorts may reveal novel targets and pathways for preventative therapies.

Lower limb fracture and orthopaedic surgery are well-established risk factors of venous thromboembolism (VTE), driven by vascular injury, inflammation, and hypercoagulability. Indeed, post-fracture DVT remains a significant clinical challenge, with incidence rates reaching up to 60% in specific fracture populations [35]. Recent studies have revealed that the proteomic profiles of trauma patients who developed DVT are characterised by a complex interplay of systemic coagulation and complement dysregulation, amplified inflammation, and local tissue stress responses. Mohammed et al. [36] evaluated targeted quantitative proteomics in two distinct provoked VTE cohorts derived from the patients with knee arthroscopy (POT-KAST) and lower-leg cast immobilisation (POT-CAST). In the POT-KAST cohort, future VTE cases showed lower APOC2, APOC3, APOC4, ATRN, Factor XIIIB and prothrombin, while the POT-CAST cohort was characterised by higher APOB, APOE, SERPINF2, B2M, AFM and Factor XIIIB. However, the sampling time point relative to trauma and thrombosis differed between the cohorts. In POT-CAST, blood was collected after lower-leg injury, whereas in POT-KAST it was collected before arthroscopy. Therefore, clinical interpretation about the divergent findings needs to be cautious because it may represent different biological states, with the POT-CAST cohort likely also capturing a post-traumatic systemic pro-coagulant response, rather than just a single uniform provoked VTE signature. Zhang et al. [37] showed that besides significantly altered pathways of complement and coagulation, upregulation of glycolysis/gluconeogenesis proteins (LDHA, LDHB, PKM) were observed in DVT patients with acute traumatic fractures, suggesting a metabolic shift that may promote thrombosis.

Other patient populations with known prothrombotic risk factors such as active autoimmune disease [38] and obesity [39] have also been investigated. Weitz et al. [38] used proteomics to try to account for the increased VTE incidence seen in tofacitinib-treated rheumatoid arthritis patients. No single biomarker fully accounted for the excess VTE risk in the tofacitinib arm. For patients with overweight/obesity, which are known risk factors for VTE, Ten Cate and Koek et al. [39] found that an 11-protein signature was associated with body mass across both the acute phase following VTE diagnosis and 12-month follow-up. However, this signature was not associated with VTE outcomes and importantly could not explain the controversial ‘obesity-paradox’, whereby obese individuals exhibit paradoxically lower rates of recurrent VTE, and mortality compared to those with normal BMI [40]. This study [39], however, did find that leptin was inversely associated with recurrent VTE or death (adjusted HR [95% confidence interval] per standard deviation increase: 0.66 [0.46–0.94]) and was modified by markers of leptin resistance).

**Table 1:** Summary of extracted studies.

Included Studies	Cohort	Sample Collection Time Point	Sample Type	Proteomic Methods	Key Findings	
					Proteomic Findings	Clinical Significance of Candidate Proteins
VTE patients vs. non-VTE controls						
Pre-VTE development						
Jensen et al. 2018 [11]	Nested case–control study. All participants in this study came from the 4th Tromsø Study cohort (n = 27,158). 1st lifetime VTE Patients (n = 80) DVT: n = 55 PE: n = 25 Non-VTE Control (n = 88)	Median: 3.8 years (Range: 0.09–6.85) before VTE	EDTA plasma	Untargeted LC-TMT-SPS-MS/MS/MS	In total, 681 proteins were identified; 46 proteins were identified as potential biomarker candidates for VTE prediction. The strongest predictive biomarkers were transthyretin, ProZ, and DJ-1.	TTR: Promotes inflammation by transporting Thyroxine ProZ: Elevated levels reflect pro-coagulant status DJ-1: Suggests oxidative stress and protein repair mechanisms in VTE Factor IX: Promotes thrombosis Gal3BP: Involved in platelet activation and inflammation Calprotectin: Involved in neutrophil activation and inflammation
Jensen et al. 2022 [17]	Follow-up study of Jensen et al. 2018 [11]. Cohort in this study were also all came from the 4th Tromsø Study cohort (n = 27,158). 1st lifetime VTE Patients (n = 410) DVT: n = 253 PE: n = 157 Non-VTE Control (n = 834)	DVT: Mean: 6.60 years (Range: 0.09–12.80) before DVT event PE: Mean: 8.10 years (Range: 0.65–12.60) before PE event	EDTA plasma	Targeted ELISA	The re-analysis of the proteomic dataset from the 2018 study [11] identified LBP as a candidate biomarker. The ELISA in this study further validated elevated LBP as a predictive DVT biomarker in women with less than 3 years between blood sampling and DVT.	LBP: An acute-phase protein that binds LPS and promotes immune activation.
Li et al. 2023 [19]	Primary GWAS cohort: UK biobank VTE Patients (n = 14,429) Non-VTE Control (n = 267,037) Proteomic reference cohort: ARIC study Non-VTE Control (n = 7213) Validation cohort: GSE19151 dataset VTE Patients (n = 47) Healthy Control (n = 48)	Before VTE, but did not specify the exact timepoint	Retrospective data analysis of published proteomic data	Targeted SomaScan v4.1	In total, 20 plasma proteins (Factor II, Factor XI, ABO, PLCG2, LRP4, PLEK, KLKB1, PROC, KNG1, THBS2, SERPINA1, RARRES2, CEL, GP6, SERPINE2, SERPINA10, OBP2B, EFEMP1, F5, and MSR1) were identified as associated with VTE. Specifically, 13 proteins showed causal relationships with VTE, including Factor II, Factor XI, ABO, PLCG2, LRP4, PLEK, PROC, KNG1, THBS2, SERPINA1, SERPINE2, SERPINA10, EFEMP1.	Pathway analysis of candidate proteins (20 proteins): Factor II, Factor V, Factor XI, KLKB1, PROC, KNG1, SERPINA1, SERPINE2, SERPINA10: Involved in Complement and coagulation cascades. Factor II, PLCG2, PLEK, GP6, SERPINE2: Involved in platelet activation. Factor II and RARRES2: Involved in immune response.
Yuan et al. 2023 [23]	Swedish Cohort VTE (n = 352) Non-VTE Control (n = 11,451) MVP+UK Biobank VTE (n = 26,066) Non-VTE Control (n = 624,053) FinnGen Cohort VTE (n = 14,454) Non-VTE Control (n = 294,700)	Median: 6.6 years before VTE	Unspecified	Targeted Olink proximity extension assay	In total, 257 proteins were identified; 21 proteins were associated with incident VTE. MR analysis indicated causal associations of vWF, PAI-1, EPHB4, TYRO3, TNFRSF11A, BOC and CHI3L1 with VTE risk	vWF: Related to Factor VIII and promote platelet adhesion and coagulation. PAI-1: Related to fibrinolysis EPHB4: Related to vascular morphogenesis. TYRO3: Related to platelet aggregation. TNFRSF11A: Related to inflammation.
Yuan et al. 2024 [21]	Primary GWAS cohort: Icelanders General participants (n = 35,559) Replication cohorts: Fenland study general participants: (n = 10,708) UKB-PPP general participants (n = 54,219) VTE cohort VTE Patients (n = 81,190) Non-VTE Control (n = 1,419,671)	Before VTE, but did not specify the exact timepoint	Retrospective data analysis of published proteomic data	Targeted SomaScan v4	In total, 34 proteins were significantly associated with VTE. Specifically, 23 proteins showed causal relationships with VTE. Notably, FXI mediated up to 40% of the smoking–VTE association and 27% of the insomnia–VTE association. There were 12 identified proteins already targeted by approved drugs, 5 proteins were associated with both VTE and cardiovascular diseases.	Pathway analysis of candidate proteins (20 proteins): Factor XI, Factor II, Factor XII, Protein S: Involved in coagulation cascades. IL-6, ANGPT1: Involved in inflammation. LRP12, LRP4: Involved in lipid metabolism and endothelial function.
Kong et al. 2025 [22]	Primary GWAS cohort: UK biobank VTE Patients (n = 22,103) Non-VTE Control (n = 356,371) PWAS cohort: UKB-PPP cohort VTE Patients (n = 768) Non-VTE Control (n = 27,550)	Before VTE, but did not specify the exact timepoint	Retrospective data analysis of published proteomic data	Targeted SomaScan v4.1	In total, 64 proteins showed significant linear association, and 43 proteins showed significant non-linear associations with VTE. Specifically, 8 proteins showed similar dose–response curves in both PWAS and cohort replication: ULBP2, IL18BP, MAN1A2, CCL25, ICAM2, LGALS4, VSIG2, and ABO.	Pathway analysis of different expressed proteins: For the 43 non-linear VTE-associated proteins: enriched pathway includes endothelium development, fluid shear stress and atherosclerosis For the 64 linear VTE-associated proteins: enriched pathway includes blood coagulation, body fluid regulation, focal adhesion, Rap1 and Ras signalling,
Post-VTE: acute phase (at presentation or within 2 weeks after diagnosis)						
Memon et al. 2018 [12]	Patients were selected from the SCORE study cohort (n = 357) DVT Patients (n = 45) Non-DVT Controls (n = 45)	At the time of suspected DVT presentation	Citrated plasma	Targeted Olink proximity extension assay	In total, 92 proteins were identified; 30 proteins showed significant differences between DVT and non-DVT patients. After Bonferroni correction, plasma levels of seven proteins, SELP, TfR1, vWF, TFPI, OPN, BLMH and ST2, were increased in DVT patients.	SELP, TFPI, and vWF: Promote platelet adhesion and coagulation OPN: Involved in inflammation and coagulation. ST2: Involved in tissue factor induction and endothelial activation. TfR1: Indicates relationship between iron metabolism and thrombosis risk. BLMH: Indicates relationship between homocysteine metabolism and vascular complications
Zhang et al. 2018 [14]	Acute PE Patient (n = 24) Healthy Control (n = 24)	After diagnosis of acute PE	Serum	Untargeted 2D-DIGE, MALDI-TOF-MS	In total, 8 proteins (TRY-3, HP, LRG, CLUS, AMBP, ITIH4, K1C9, RET4) were identified to cause a significant increase in the serum of acute PE patients. HP was highlighted as the most promising biomarker.	HP: Reduces oxidative damage; increased level indicates acute-phase reaction and thrombus-associated inflammation in PE.
Han et al. 2021[15]	Discovery study Non-high-risk PE (n = 6) High-risk PE (n = 3) Healthy Control (n = 4) Antibody array study Non-high-risk PE (n = 10) High-risk PE (n = 10) Healthy Control (n = 12) Verification study Non-high-risk PE (n = 25) High-risk PE (n = 25) Healthy Control (n = 26)	Within 24 h of admission and after heparin treatment	Citrated plasma	Untargeted DIA-LC-ESI-MS/MS & Targeted label-based antibody array, ELISA	In total, 1161 proteins were identified by DIA-MS and biotin label-based antibody array. PE patients vs. healthy controls: 51 proteins were increased, and 166 proteins were decreased in PE patients Among all comparisons, 16 proteins had significant differences across all groups, including IGLV3-19, IGKV1D-39, IGKV4-1, IGHA1, FETUB, PLG, ICAM2, ACTN1, SAA4, AMBP, HRG, F13B, HP, TF, TNC, and ORM1.	1. Pathway analysis of different expressed proteins: For PE vs. healthy (217 proteins): enriched pathways included complement activation, response to wounding, HDL particle remodelling, and negative regulation of peptidase activity. 2. Clinical significance of candidate proteins: SAA1 and S100A8: Promote inflammation by leukocyte recruitment and cytokine secretion. TNC: Involved in tissue repair and wound healing. GSN: Inhibits inflammation and could be potential therapeutic target. HRG: Inhibits excessive clot formation by interfering with contact activation
Granholm et al. 2022 [16]	Male PE patients (n = 8) Male non-PE patients (n = 8)	On admission and after 12 months of anticoagulant therapy	Citrated plasma	Untargeted LC-ESI- MS/MS	In total, 137 proteins were identified, 3 proteins (C9, CFH, and LRG1) were increased, and 10 proteins (ApoC-III, CEH, ATIII, PCPE, SERPINA4, CPB2, TAFI, AFM, and SERPINA5) were decreased in PE patients.	C9, and CFH: Involved in complement, inflammation and thrombosis. LRG1: Promote angiogenesis and inflammation. ATIII, TAFI: Inhibits thrombosis by inhibiting thrombin. SERPINA4 and 5: Inhibit coagulation and inflammation. CPB2: Inhibits fibrinolysis
Iglesias et al. 2023 [18]	VEBIO Discovery study VTE Patients (n = 48) Non-VTE Control (n = 48) VEBIO validation study VTE Patients (n = 144) Non-VTE Control (n = 140) DFW-VTE study (Sweden) VTE Patients (n = 54) Non-VTE Control (n = 146) FARIVE study (France) VTE Patients (n = 582) Non-VTE Control (n = 576) RETROVE study (Spain) VTE Patients (n = 308) Non-VTE Control (n = 360) MARTHA study (France) VTE Patients (n = 1322) With 699 recurrence follow-up	VEBIOS discovery: On admission, before anticoagulant therapy. VEBIOS coagulation: 1–6 months after stopping anticoagulant therapy. FARIVE: Within 1 week after VTE diagnosis, during initiation of treatment. RETROVE: 6–12 months after anticoagulant therapy MARTHA: On admission Long-term follow-up for recurrence.	Citrated and EDTA plasma	Targeted bead array, IC-MS, & Untargeted DIA-MS	CFHR5 is associated with VTE and has potential application as a clinical biomarker for VTE diagnosis and risk prediction. Higher CFHR5 levels are associated with increased thrombin generation and recombinant CFHR5 enhanced platelet activation in vitro.	Pathway analysis showed CFHR5 clusters with complement pathway genes, enriched in pathways of Complement activation, regulation of complement cascade, and Humoral immune response.
Wang et al. 2024 [20]	Biomarker discovery stage: DVT (n = 10) Non-lower extremity DVT (n = 10) Healthy Control (n = 5) Biomarker validation stage: Included all participants from discovery stage and additional participants DVT (n = 15) Non-lower extremity DVT (n = 15) Healthy Control (n = 6)	Within 3 days of disease onset	Citrate plasma	Targeted antibody arrays, ELISA	In total, 59 proteins were found to be differentially expressed between the DVT and healthy control, 50 proteins were increased, and 9 proteins were decreased in DVT group. Validation study confirmed 12 biomarkers (EDA-A2, FGF-6, Dkk-4, IL-1 Factor IX, Gal-3, LYN, bIG-H3, ULBP-2, Gas-1, IGFBP-5, and FGF-9) were significantly upregulated in DVT group.	1. Pathway analysis of different expressed proteins (59 proteins between the DVT and healthy control groups) GO analysis: Enriched pathways included responses to molecules of bacterial origin, response to lipopolysaccharide, positive regulation of cytokine production, peptidyl-tyrosine modification, neutrophil migration, neutrophil chemotaxis, leukocyte migration, and leukocyte chemotaxis. KEGG pathways: Enriched pathways included JAK-STAT signalling, IL-17 signalling, cytokine–cytokine receptor interaction, infection-related pathways, Viral protein interaction with cytokines and cytokine receptors, infection and inflammation, and Melanoma pathway 2. Clinical significance of candidate proteins: Gal-3 and CHI3L1: Promote inflammation by enhancing endothelial cell migration and angiogenesis. IL-1, Factor IX: Promote inflammation by triggers NF-κB and MAPK signalling and inducing cytokine production.
Zhuang et al. 2025 [24]	Discovery proteomics: VTE (n = 3) Healthy Control (n = 3) ELISA validation: VTE (n = 17) Healthy Control (n = 17)	On admission, before anticoagulant therapy.	Citrate plasma	Untargeted iTRAQ-LC-MS/MS & Targeted ELISA	In total, 72 proteins were found to be differentially expressed between the VTE and healthy control, 44 proteins were increased, and 28 proteins were decreased in VTE group. ELISA validation study confirmed HPX was increased in VTE group	Pathway analysis of 72 differentially expressed proteins showed enrichment in complement and coagulation cascades
Post-VTE—subacute phase						
Flores-Nascimento et al. 2012 [9]	DVT Patients (n = 3) Non-DVT Control (n = 6) For each patient, two controls were included One sibling (As genetic control) One neighbour (As environmental control)	Median: 2 years (Range 1–4 years) after DVT event	EDTA plasma	Untargeted LC-nanoESI-Ion Trap MS/MS	In total, 199 proteins were identified, 4 proteins (C4A, ITIL, ITIH1, and SAA) were increased and 3 proteins (AHSG, ITIH4, and APOA4) were decreased in DVT patients.	C4A: Involves in complement pathway activation. ITIL, ITIH1, and SAA: Promote inflammation. AHSG: Inhibits vascular inflammation. APOA4: Inhibits platelet aggregation and thrombus formation.
Bruzelius et al. 2016 [10]	VEBIOS discovery study: VTE Patients (n = 88) Non-VTE Control (n = 85) FARIVE verification study VTE Patients (n = 580) Non-VTE Control (n = 589)	VEBIOS discovery: 1–6 months after stopping anticoagulant therapy. FARIVE Verification: Within 1 week after VTE diagnosis, during initiation of treatment.	Citrated and EDTA plasma	Targeted IC-MS, ELISA	In total, 408 proteins were identified, 3 proteins (PDGFB, VWF, HIVEP1) were increased and 1 protein (GPX3) was decreased in VTE patients.	PDGFB: Promote thrombosis by promoting endothelial and platelet activation. VWF: Promote platelet adhesion and coagulation HIVEP1: Involved in inflammation. GPX3: Inhibit thrombosis by reducing oxidative stress.
Proteomics to study differences between different subtypes of VTE						
Insenser et al. 2014 [34]	Discovery study Acute PE patients (n = 12) High/Inter- risk: n = 6 Low risk: n = 6 Validation study Acute PE patient (n = 104) High/Inter- risk: n = 52 Low risk: n = 52	Within 24 h of diagnosis	EDTA plasma and serum	Untargeted 2D-DIGE, LC-MALDI-TOF/TOF MS/MS & Targeted ELISA	In discovery study, 4 proteins were differentially expressed between low-risk and intermediate/high-risk PE patients. Haptoglobin and Hemopexin were decreased, while A2M and IgA1 were increased in intermediate/high-risk PE patients	Haptoglobin and Hemopexin: Involves in iron metabolism and haemolysis, protects against oxidative stress and endothelial damage. A2M: Promotes thrombosis by Inhibiting fibrinolysis. IgA1: Promotes immune activation.
Han et al. 2021[15]	Discovery study Non-high-risk PE (n = 6) High-risk PE (n = 3) Healthy Control (n = 4) Antibody array study Non-high-risk PE (n = 10) High-risk PE (n = 10) Healthy Control (n = 12) Verification study Non-high-risk PE (n = 25) High-risk PE (n = 25) Healthy Control (n = 26)	Within 24 h of admission and after heparin treatment	Citrated plasma	Untargeted DIA-LC-ESI-MS/MS & Targeted label-based antibody array, ELISA	In total, 1161 proteins were identified by DIA-MS and ELISA. High-risk vs. non-high-risk PE patients: 45 proteins were increased, and 25 proteins were decreased in high-risk PE patients. Among all comparisons, 16 proteins had significant differences across all groups, including IGLV3-19, IGKV1D-39, IGKV4-1, IGHA1, FETUB, PLG, ICAM2, ACTN1, SAA4, AMBP, HRG, F13B, HP, TF, TNC, and ORM1.	1. Pathway analysis of different expressed proteins: For high-risk vs. non-high-risk PE (70 proteins): Enriched pathways included regulation of blood coagulation, acute inflammatory response, positive regulation of chemokine production, and receptor-mediated Endocytosis. 2. Clinical significance of candidate proteins: SAA1 and S100A8: Promote inflammation by leukocyte recruitment and cytokine secretion. TNC: Involved in tissue repair and wound healing. GSN: Inhibits inflammation and could be a potential therapeutic target. HRG: Inhibits excessive clot formation by interfering with contact activation
Razzaq et al. 2021 [33]	MARTHA Patients cohort (n = 1388) DVT: n = 1105 PE: n = 95 DVT+PE: n = 188	On admission	Plasma	Targeted multiplex antibody suspension bead array	Top 20 most contributing antibodies/proteins of the definition of the LIME predictor were identified. Specifically, 5 proteins tended to have substantial more importance than the remaining ones, including VCL, COX412, FABP6, ROCK1, and VWF. Based on artificial neural network, ANN model was more accurate in predicting DVT (ANN: 0.872 vs. LIME: 0.748), while LIME model appears slightly more accurate in predicting PE (ANN: 0.498 vs. LIME: 0.578).	VCL, VWF, ROCK1: Promote platelet adhesion and coagulation COX4I2: Promotes inflammation by increasing mitochondrial reactive oxygen species FABP6: Links lipid metabolism to inflammatory signalling
Ten cate and Prochaska et al. 2021 [31]	GMP-VTE project VTE patients (n = 532) DVT: n = 160 PE: n = 96 DVT+PE: n = 276	At the time of indication for diagnostic imaging or shortly after imaging had confirmed the diagnosis	EDTA plasma	Targeted Olink proximity extension assay	IFN-γ, GALNT3, and β-NGF were among the proteins with the highest expression within the isolated PE phenotype, while CTSD, IDUA and PAI-1 were the proteins with the lowest expression in the isolated PE phenotype. In the isolated PE vs. isolated DVT model, 56 proteins were selected, providing an AUC of 0.96. In the isolated PE vs. DVT+PE model, 18 proteins were selected, achieving an AUC of 0.76. In the DVT+PE vs. isolated DVT model, 61 proteins were selected, showing an AUC of 0.89.	1. STRING Pathway analysis Proteins of top 10% highest/lowest in isolated PE: clusters in networks included Immunoregulatory, neurotrophin, tissue remodelling, Surfactant/lung-specific Proteins highly correlated: clusters in networks included Immunoregulatory, tissue remodelling and tissue repair 2. Clinical significance of candidate proteins: FN-γ and IL-15Ra: Promote inflammation by promoting Th1 immune response. GDNF: Promotes neurotrophin signalling. GALNT3: Inhibits inflammation by inhibit NF-κB pathway. PADI2: Promotes inflammation by promoting neutrophil extracellular traps (NETs)
Baidildinova et al. 2022 [32]	GMP-VTE project VTE patients (n = 541) DVT: n = 160 PE: n = 99 DVT+PE: n = 282	At the time of indication for diagnostic imaging or shortly after imaging had confirmed the diagnosis	EDTA plasma	Targeted Olink proximity extension assay	In total, 135 proteins were identified as platelet-related proteins to distinguish PE, PE+DVT from DVT only. Specifically, 33 proteins identified to distinguish isolated PE from isolated DVT, with 20 proteins increased and 13 proteins decreased in isolated PE patients. Meanwhile, 30 proteins were identified to distinguish PE+DVT from isolated DVT, with 13 proteins increased and 17 proteins decreased in PE+DVT patients. In addition, 11 proteins common to both PE subtypes, but with inverse expression directions between subtypes were identified.	STRING Pathway analysis The 20 upregulated proteins of isolated PE when compared to isolated DVT: clusters in networks included immune receptor signalling, pathogen clearance, and chemotaxis The 19 proteins that specifically regulated and unique in PE+DVT: clusters in networks included tissue remodelling and leukocyte trafficking.
Wang et al. 2024 [20]	Biomarker discovery stage: DVT (n = 10) Non-lower extremity DVT (n = 10) Healthy Control (n = 5) Biomarker validation stage: Included all participants from discovery stage and additional participants DVT (n = 15) Non-lower extremity DVT (n = 15) Healthy Control (n = 6)	Within 3 days of disease onset	Citrate plasma	Targeted antibody arrays, ELISA	In total, 62 proteins were found to be differentially expressed between the DVT and Non-lower extremity DVT groups, 48 proteins were increased, and 14 proteins were decreased in DVT group. Validation study confirmed EDA-A2, FGF-6, Dkk-4, IL-1 Factor IX, Gal-3, LYN, bIG-H3, ULBP-2, Gas-1, IGFBP-5, and FGF-9 were promising biomarkers, demonstrating strong sensitivity and specificity for distinguishing DVT from Non-lower extremity DVT.	Pathway analysis of proteins between the DVT and Non-lower extremity DVT) GO analysis: Enriched pathways included response to molecules of bacterial origin and lipopolysaccharide, positive regulation of cytokine production, peptidyl-tyrosine modification, and leukocyte migration and chemotaxis KEGG analysis: Enriched pathways included Cytokine–cytokine receptor interaction, JAK-STAT signalling, IL-17 signalling, infection, and Melanoma and viral protein interaction
Proteomics in patients with different provoking factors for VTE						
Ten cate and Koeck et al. 2021 [39]	Derived from the GMP-VTE project (n = 693): VTE Patients (n = 657) Normal weight: n = 181 Overweight: n = 239 Obese: n = 237	At the time of indication for diagnostic imaging or shortly after imaging had confirmed the diagnosis and at the 12-month follow-up	EDTA plasma	Targeted Olink proximity extension assay	Obesity paradox: Normal weight individuals had the highest rates of recurrent VTE or death, while overweight individuals had a slightly reduced rate, and obese individuals had a strongly reduced incidence of recurrent VTE or death. In total, 11 proteins (CLEC4C, FABP4, FLT3LG, IL-17C, LEP, LYVE1, MASP1, IL1RL1, THBS2, THBS4, TSLP) were identified as related to BMI in VTE patients; however, the obesity paradox did not appear to be mediated by the proteomic signature. Only LEP was significantly associated with recurrent VTE or death.	LEP: Protective effect by endothelial nitric oxide synthase activation, promoting vascular health
Weitz et al. 2022 [38]	Derived from the ORAL Surveillance trial (n = 4362): Patients with active rheumatoid arthritis (n = 285) Patients with VTE: n = 57 Patients without VTE: n = 228	On baseline and after 12 months of randomised treatment of tofacitinib, and adalimumab	Serum and Plasma	Targeted Olink proximity extension assay	On Month 12, higher levels of D-dimer and TPO were associated with a greater risk of a subsequent VTE events in patients with tofacitinib treatment. ANG and TNFSF13B showed significant associations with PE events in patients with tofacitinib treatment.	TPO: Promotes platelet activation via JAK2 signalling. ANG and TNFSF13B: Involved in immune regulation, angiogenesis and vascular remodelling
Mohammed et al. 2022 [36]	Derived from the POT-KAST trial (n = 1500) Patients for knee arthroscopy (n = 24) Patients with VTE: n = 8 Patients without VTE: n = 16 Derived from the POT-CAST trial (n = 1500) Patients for lower-leg cast immobilisation (n = 69) Patients with VTE: n = 23 Patients without VTE: n = 46	Patients for knee arthroscopy: within 4 h before surgery (Pre-trauma) Patients for lower-leg cast immobilisation: on admission (Post-trauma)	Citrated and EDTA plasma	Targeted LC-ESI-MS/MS	In total, 167 proteins were quantified. Specifically, APOC3, ATRN, APOC4, APOC2, F13B, and F2 were decreased in patients with VTE after knee arthroscopy, while increased APOB, APOE, A2AP, B2M, AFM, F13B, and decreased C1QC were observed in patients with VTE after lower-leg cast immobilisation.	Apolipoproteins (APOC2, APOC3, APOC4, APOE, APOB): Involved in lipid metabolism and triglyceride-rich lipoproteins. Coagulation factors (Factor II, XIII): Promote thrombosis and involved in coagulation cascades. A2AP: Inhibits fibrinolysis by promoting clot persistence. C1QC and ATRN: Involved in immune system activation and inflammation.
Zhang et al. 2024 [37]	Derived from patients with acute traumatic fractures (n= 580) DVT patients (n = 96) Non-DVT controls (n = 87)	On admission	Plasma	Untargeted LC-ESI- MS/MS	In proteomics, 524 proteins were identified, 214 proteins showed significant difference, with 153 upregulated and 61 downregulated in DVT. Specifically, 8 proteins (LDHA, LDHB, GAPDH, GPI, PKM, MINPP1, ENO1, and TPI1) were upstream of pyruvic acid, indicating upregulation of glycolysis/gluconeogenesis and TCA cycle cascaded.	1. KEGG pathway analysis of the 214 differential proteins: In total, 15 significantly altered pathways, with the most notable pathways involve Complement and coagulation cascades, TCA cycle, and Glycolysis/gluconeogenesis 2. Clinical significance of candidate proteins: LDHA and LDHB: Key glycolytic enzymes; linked to ROS production. PRDX5 and PRDX2: Redox regulators, involved in oxidative stress in RBC and promoting platelet activation. Complement factors of C3, C4, C5: Central proteins in complement activation.
Other studies						
Bryk-Wiązania et al. 2022 [30]	Acute PE patients (n = 5)	On admission and after 3 months of rivaroxaban treatment	Pooled citrated plasma and purified plasminogen	Untargeted LC-ESI-MS/MS	In total, 44 proteins were identified both at baseline and after 3 months from the pooled plasma, including fibrinogen chains, A2AP, A2M, ITIL, and others. The plasminogen samples revealed 88 different post-translational modifications, occupying 162 sites. Specifically, 20 sites of oxidation, 3 sites of carbonylation and 4 sites of S-nitrosylation were identified in the purified plasminogen.	Post-translational modifications of plasminogen: Oxidation of cysteine residues may alter plasminogen conformation and impair fibrinolysis Carbonylation and nitrosylation linked to oxidative stress and hypo-fibrinolysis. Modifications at active sites may affect activation and fibrin interaction.
Pallares Robles et al. 2022 [29]	Derived from the GMP-VTE project VTE patients (n = 549) Low FXI activity: n = 43 Medium: n = 412 High: n = 94	During the acute phase of VTE and at the 12-month follow-up	EDTA plasma	Targeted Olink proximity extension assay	Factor XI activity was significantly higher in the acute phase of VTE than that of 12-month follow-up. Among the 444 proteins incorporated in the present analysis, 21 proteins and 66 proteins were associated with Factor XI activity for the acute VTE event and at 12 months follow-up, respectively. These proteins were involved in multiple pathways.	KEGG Pathway analysis of FXI-associated proteins Enriched pathways in both time points: IL-1 signalling, apoptosis, TNFR2 signalling, NF-κβ signalling, TNF/stress-related signalling, TRAF6 induction of NF-κβ and MAP kinases upon Toll-like receptor, activated TLR4 signalling and signal transduction through IL1R Enriched pathways in acute phase only: B cell receptor signalling pathway and extracellular matrix interaction Enriched pathways in 12-month only: Neurotrophin signalling, fibroblast growth factor receptor signalling, signalling by interleukins, Toll receptor cascades, MAPK signalling pathway, RIG-I-like receptor signalling pathway, metabolism of lipids and lipoproteins, cytokine signalling in immune system and NOD-like receptor signalling

Proteomic studies of provoked VTE cohorts, triggered by factors like trauma or surgery, reveal a complex immunothrombotic milieu characterised by complement and coagulation dysregulation. After trauma or orthopaedic surgery, patients who develop DVT show elevated complement and coagulation proteins alongside inflammation and metabolic changes, reflecting an “immunothrombotic” state. Immobilisation-related VTE is associated with increased Factor XIII and altered apolipoproteins, while patients with Achilles tendon rupture further demonstrate suppressed extracellular matrix proteins and active hypoxic responses during the inflammatory healing phase. These studies advance our mechanistic understandings of VTE development following these provoking factors; however, utilising these findings in clinical application remains not yet achieved.

## 4. Discussion

In this narrative review, we have summarised the current proteomic studies spanning the full VTE continuum, including pre-event risk cohorts, acute VTE, convalescent-phase, different VTE phenotypes, and selected provoked VTE cohorts. These studies have significantly advanced our understanding of the proteomic landscape of VTE. A unifying thromboinflammation proteomic profile has been presented from years before VTE, through the acute event, and toward the convalescence phase, despite considerable heterogeneity in cohort design, sampling time point, sample type, and analytical platform. However, the literature remains largely exploratory, and few candidates have progressed beyond discovery into robust external validation or clinically meaningful implementation.

### 4.1. Proteome Characteristics: Thromboinflammation in VTE

**Figure 2:**
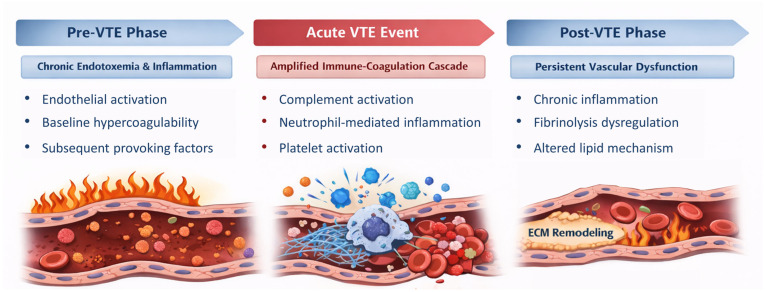
Thromboinflammation in VTE.

The consistent emergence of inflammatory and complement-driven signatures across the VTE continuum revealed that VTE pathogenesis involves a continuous, maladaptive cycle of thromboinflammation, rather than just a localised thrombosis event. from years before VTE, low-grade, chronic endotoxemia and inflammation could activate the endothelium and create a baseline hypercoagulable state [17,19]. This pre-existing priming likely lowers the pathophysiological threshold for thrombosis, meaning that subsequent provoking factors—such as trauma or orthopaedic surgery—can easily trigger an exaggerated, systemic immune-coagulation response. During the acute phase, this pre-existing state culminates in an intense, amplified cascade characterised by rampant complement activation [15,16,18], neutrophil-mediated inflammation and rapid platelet activation [12,14,15,16,28]. Distinct VTE phenotypes appear to present varied acute thromboinflammatory phenotypes [31,32,33], with high-risk PE demonstrating profound hemodynamic stress and aggressive acute-phase responses leading to severe haemolysis, whereas isolated DVT exhibits a more localised immune-endothelial interaction. Following the acute event, the proteomic landscape does not simply return to homeostasis. Instead, convalescent-phase profiling reveals a shift toward sustained fibrinolysis perturbation, extracellular matrix turnover, altered lipid metabolism, and chronic inflammatory signalling, which suggests that the initial thromboinflammatory burst leaves a persistent “immunological scar” on the vasculature, fostering prolonged vascular remodelling, risk of recurrent VTE and other potential post-thrombotic complications [9,10] (Figure 2).

While the thromboinflammatory interpretation of these findings is biologically plausible, it must be applied cautiously. Current VTE proteomics studies support thromboinflammation as a recurring biological pattern in VTE, but it does not yet definitively define which proteins are true causal mediators, which are downstream epiphenomena, and which could ultimately serve as the potential biomarkers for clinical use. Furthermore, the clinical application of these candidate biomarkers is currently hindered by significant translation barriers inherent in the included studies.

### 4.2. Barriers to Clinical Translation of Proteomic Findings (Table 2)

A key methodological challenge in VTE proteomics is the choice of sample type. Most studies to date have used either plasma or serum. However, as serum preparation requires activation of the coagulation cascade, this results in the significant depletion of fibrinogen and other key coagulation proteins. Consequently, the residual coagulation proteins in serum are not able not reflect their accurate physiological levels or functional states. The following proteomic analyses such as protein identification, pathway enrichment, or protein–protein interaction networks reflect only the residual post-clotting proteins and are inherently biased [41]. Additionally, additives to the plasma sample can also introduce important variability. Mohammed et al. [36] demonstrated that citrated plasma yields lower median protein concentrations compared to EDTA plasma, and some proteins were detectable only in EDTA. Despite these differences, relative protein trends correlated well between EDTA and citrated plasma. EDTA is optimal for proteomic discovery because it serves as a stronger chelator, effectively inhibiting intrinsic protease activity and preserving protein integrity, while the weaker inhibition by citrate may allow for partial degradation of labile proteins [42]. However, EDTA irreversibly chelates metal ions required for coagulation; thus, samples are not suitable for further functional clotting assays, which is a practical limitation when both proteomics and functional testings such as global coagulation assays are required. Therefore, researchers need to carefully consider the need for proteomic stability against the potential requirement for functional validation, for which citrate remains the standard.

**Table 2:** Barriers to clinical translation of proteomic findings in VTE.

Barriers		Impact on Findings and Clinical Translation
Sample Type	Plasma vs. serum	Serum preparation depleted fibrinogen and other key coagulation proteins, limiting comparability and reducing biological validity
	Different plasma anticoagulants (EDTA vs. citrate)	EDTA plasma showed higher detectability and concentration, but not suitable for further functional clotting assays
Sampling Timepoints	Heterogeneous sampling timepoints (Pre- vs. Acute vs. Post-VTE)	Different sampling time points showed distinct proteomic signatures, reflecting different aspects of the pathophysiology of VTE
	Lack of VTE recurrence studies	Lack of proteomic data to identify patients with high VTE recurrence risk
Analytical Workflow	Different proteomic platforms (Untargeted vs. targeted proteomics)	Untargeted proteomics offers unbiased and comprehensive proteome profiling but requires complex instrumentation and sample preparations. Targeted proteomics offers higher sensitivity but are restricted to pre-selected protein panels
	Sample processing and instrument variability	Different pre-analytical and analytical workflows of proteomic analysis result in inconsistent findings
	Inter-lab variability	Varied sample preparation and instrumental settings between labs attribute to inconsistent findings
Statistical Analysis	Heterogeneous statistical criteria	Different statistical criteria result in different proteins being identified with inconsistent effect sizes

Among the included studies in this review, the sampling time points ranged from years before the development of VTE to long-term follow-up after VTE events. The optimal timing point for proteomic studies are clearly not yet standardised and may be a result of the practical constraints of available biobanks. Due to the dynamic haemostatic changes in VTE patients, pre-event, acute and post-event proteomes differ greatly. Studies collecting samples at varied time points are interrogating different aspects of the pathophysiology of VTE. Pre-VTE proteomic signatures are more informative for primary prevention, whereas acute-phase proteomic signatures may be useful for diagnosis. Proteomic markers identified from post-VTE samples are relevant for identifying patients who remain in a high-risk, prothrombotic state and who might benefit from anticoagulation therapy or targeted follow-up. However, in this review, we have not identified any studies which has specifically examined proteomic differences in VTE patients who have developed future recurrences. This is an important gap in the knowledge as there is currently no biomarker which can specifically identify patients at high risk of future VTE recurrence, knowledge of which may better target anticoagulation therapy in the longer-term.

The variability across proteomic platforms and the lack of standardisation in proteomic analysis workflows hinder the clinical translation of proteomic candidates in current VTE proteomics studies, leading to low reproducibility and inconsistency in findings. Studies have utilised either untargeted, mass spectrometry–based discovery approaches or targeted, affinity-based protein detection panels, and there are also substantial differences within each method, such as shotgun versus data-independent acquisition in untargeted MS, different affinity platforms such as aptamer-based assay (e.g., SomaScan), proximity extension assays and ELISA in targeted proteomic approaches. Untargeted proteomic methods offer unbiased and comprehensive profiling of the entire proteome but require complex instrumentation and complex sample preparations. Factors like instrument tuning, ionisation efficiency, peptide fragmentation and following protein identification are attributed to high variability across labs and platforms [43]. Conversely, targeted affinity-based platforms offer higher sensitivity but are restricted to pre-selected protein panels, which lacks the exploratory power when compared with untargeted proteomic approaches [44,45]. Therefore, comparing a biomarker identified via label free mass spectrometry to ones validated via antibody-based assays is often challenging, as these platforms measure protein abundance through fundamentally different physical mechanisms [46]. Even within similar approaches, sample preparation and instrumental settings can be varied significantly between labs, which attribute to the differences in protein identification. As a result, candidate biomarkers identified on one platform may not even be measurable on another, the significant enriched pathways are varied across studies, or same enriched pathway may be represented by different proteins.

In addition, pre-analytical and analytical workflows of proteomic analysis are rarely standardised among current studies. Sample preparation protocols including high-abundance proteins depletion, protein fractionation/digestion, and instrument setting differ substantially across studies. Furthermore, the statistical criteria for data normalisation and defining differently expressed proteins also vary, with some studies relying on *p*-value thresholds and others using fold-change cut-offs or more stringent false discovery rate corrections [47]. Together, these methodological differences make it difficult to compare effect sizes between studies, and proposed protein predictors in one VTE cohort could be statistically dismissed in other studies. Therefore, it is essential to standardise proteomic platforms, sample processing and statistical criteria for VTE proteomics to progress towards clinically useful biomarkers identification.

### 4.3. Future Directions

#### 4.3.1. Expanding Proteomic Studies Across Diverse VTE Cohorts

Although proteomic studies have revealed many novel VTE biomarkers to date, the pathophysiology and underlying VTE mechanisms in several high-risk populations remain largely undefined. As previously discussed, no proteomic data directly compare patients with recurrent or multiple previous VTE events to those with a single VTE episode, which may establish protein signatures of a high-risk phenotype, and provide the basis for optimising the anticoagulation and reduce long-term morbidity. Second, the differences between provoked and unprovoked VTE have not been systematically interrogated at the proteomic level. Specific proteomic insights into patients with unprovoked VTE could potentially discover novel, intrinsic biological pathways distinct from those driven by known risk factors like surgery or trauma, allowing for distinguishing potential VTE patients from normal population. In addition, paediatric VTE patients constitute another highly specialised, vulnerable population. Due to the lack of paediatric evidence which are often extrapolated from adult studies, treatment decisions remain challenging [48]. Previous proteomic studies on healthy children have demonstrated age-specific variability in the hemostatic plasma proteome [49]. These marked differences in the hemostatic proteome between adults and children suggest the need for dedicated paediatric VTE proteomic studies to further investigate the potential age-specific mechanisms of thrombosis.

Beyond expanding patient cohorts, it is essential that future VTE proteomic research includes diverse ethnic populations [50], as the epidemiology of VTE varies significantly by ethnicity. African Americans exhibit ~5-fold higher incidence than Asian populations and are more likely to present with isolated PE than DVT [51]. Genetically, Factor V Leiden and Factor II mutations are common in Caucasians, while Asian populations often present with natural anticoagulant deficiencies, such as the Japanese-specific Protein S K196E mutation [52]. Higher baseline levels of inflammatory markers and prothrombotic factors in Black populations also suggest that universal proteomic signatures may be elusive [53]. Studies that specifically examine the ethnic proteomic differences in VTE patients are lacking and will be crucial in the search for new biomarkers. Another translational challenge lies in validation. Most current studies conclude with technical validation using low-throughput methods like ELISA, which confirms the signal but does not establish clinical utility. The future direction requires a paradigm shift by applying identified protein signatures or panels into large-scale, prospective clinical trials. This final, essential translational step will be required for these proteomic advances to move from the bench to the bedside.

#### 4.3.2. Integration of Multi-Omics in Future VTE Studies

Beyond stand-alone proteomic analyses, emerging multi-omics studies suggest that integrating genetic, proteomic, metabolomic, and lipidomic data may further strengthen both pathophysiological understanding and biomarker discovery in VTE [19,21,22,54]. Among studies utilising combined genetic–proteomic approaches [19,21,22], genetic and proteomic data from large, published population-based prospective studies were integrated, and novel VTE related proteins were identified with independent MR analysis and Bayesian co-localisation. Integrated metabolomic–lipidomic studies [54] have identified plasma metabolites and lipid species as biomarkers of historical VTE and suggest that different but complementary aspects of VTE biology may not be fully revealed by genetic or proteomic studies alone. Therefore, integrating multi-omics in future studies may provide a more comprehensive understanding of VTE pathophysiology and improve biomarker discovery by helping to prioritise more robust and biologically coherent candidates for diagnosis, prognosis, and risk stratification than any single-omics approach alone.

#### 4.3.3. Application of Artificial Intelligence and Machine Learning in Future VTE Studies

Machine learning (ML), a subset of artificial intelligence, is already increasingly used in proteomics to improve analytical workflows such as spectral library generation, peptide identification, and related data-analysis tasks [55]. Beyond proteomic data processing, ML also offers a flexible framework for integrating the heterogeneous and high-dimensional datasets increasingly relevant to VTE research, including clinical variables, laboratory results, imaging, and multi-omics data [56]. In recent years, emerging ML algorithms have been applied to improve VTE risk prediction beyond traditional scoring systems; however, substantial methodological limitations and a high risk of bias remain major barriers to clinical implementation. [57,58]. Future ML-based VTE studies should therefore be developed with rigorous feature selection, independent external validation, and prospective testing.

### 4.4. Limitations

Regarding the limitations of this specific review, this is a structured narrative review informed by a systematic literature search without prospective protocol, rather than a formal systematic review with quantitative synthesis. No formal risk-of-bias assessment nor meta-analysis was performed. The included studies were highly heterogeneous with respect to study design, patient population, provoking factors, timing of sampling, specimen type, proteomic platform, and reporting strategy, which limits direct comparison across studies. Cancer-associated VTE studies were not included in this review, because of their distinct tumour-driven proteomic signatures. However, we acknowledge that this is an important area of discovery in VTE research and proteomic profiling studies will be essential to identify novel biomarkers that differ from those observed in non-cancer VTE. Platelet proteomic studies were also not included, as the present review focused specifically on plasma-based proteomic profiling rather than cellular proteomes; however, platelet proteomics represents an important future direction that may provide complementary mechanistic and biomarker insights in VTE. Another area not covered in this review is post-thrombotic syndrome (PTS), which is an important and debilitating sequelae of DVT [59]. Future proteomic studies should focus on understanding the pathophysiology of this entity and identify biomarkers to improve the prevention and treatment of this condition. Finally, this review was restricted to English-language human studies and excluded cancer-associated VTE, so relevant findings from other settings may not have been captured.

## 5. Conclusions

VTE is now recognised as a systemic immunothrombotic disorder rather than an isolated thrombosis event, and proteomic profiling has significantly advanced our understanding of the contributing mechanisms. As highlighted in this review, complex protein signatures involving complement activation, inflammation, and platelet activation are evident even years before the VTE event, and these persist across the timecourse from the acute phase to chronic convalescence. These findings suggest that proteomic biomarkers, either as panels or individually, have the potential to improve diagnosis, risk stratification and help guide clinical management beyond current strategies. However, most proteomic findings in VTE are currently at an exploratory or early validation stage. Methodological heterogeneity including the choice of sample type, sampling time point, proteomic platforms and data analysis currently limits direct clinical implementation. Future research should prioritise the standardisation of workflows and validate promising biomarkers in large-scale, prospective and longitudinal cohorts.

## Data Availability

No new data were created or analyzed in this study.

## Abbreviations

The following abbreviations are used in this manuscript:

2D-DIGE	Two-dimensional Difference Gel Electrophoresis
A2AP	α-2-antiplasmin
A2M	α-2-macroglobulin
ABI3BP	ABI Family Member 3 Binding Protein
ABO	ABO Blood Group Glycosyltransferase
ACTN1	Actin
AFM	Afamin
AHSG	α-2-HS-glycoprotein
ALDOA	Fructose-bisphosphate aldolase A
AMBP	α-1-microglobulin
ANG	Angiopoietin
ANGPT1	Angiopoietin-1
ANT3	Antithrombin
ApoA	Apolipoprotein A
APOA1	Apolipoprotein A1
APOA4	Apolipoprotein A4
APOB	Apolipoprotein B-100
ApoB-100	Apolipoprotein B-100
APOC2	Apolipoprotein C-II
APOC3	Apolipoprotein C-III
APOC4	Apolipoprotein C-IV
ApoC-III	Apolipoprotein C-III
APOE	Apolipoprotein E
APS	Antiphospholipid syndrome
ARA	Arachidonic acid
ARIC	Atherosclerosis Risk in Communities
ATIII	Antithrombin III
ATP5B	ATP synthase subunit β, mitochondrial
ATR	Achilles tendon rupture
ATRN	Attractin
AUC	Area under the curve
B2 M	Beta-2-microglobulin
BLMH	Bleomycin hydrolase
BOC	Brother of CDO (Cell adhesion molecule-related/down-regulated by oncogenes)
C1-INH	Plasma protease C1 inhibitor
C1QC	Complement C1q subcomponent subunit C
C2	Complement component C2
C4A	Complement C4-A
C8G	Complement Component 8 Gamma Chain
C9	Complement component 9
CCL25	C-C motif chemokine ligand 25
CEH	Carboxylic ester hydrolase capillary
CE-MS	Electrophoresis coupled to mass spectrometry
CFH	Complement factor H
CFHR5	Complement Factor H Related 5 protein
CFI	Complement factor I
CFL1	Cofilin-1
CFP	Complement factor properdin
CHI3L1	Chitinase-3-like protein 1
CLEC4C	C-type lectin domain family 4 member C
CLUS	Clusterin
CO1A1	collagen type I
CO3A1	collagen type III
COX4I2	Cytochrome c oxidase subunit 4I2
TAFI	Thrombin-activatable fibrinolysis inhibitor (Carboxypeptidase B2)
CRP	C-reactive protein
CTSD	Cathepsin D
DFW-VTE study	Swedish Karolinska Age Adjusted D-dimer study
DIA	Data-independent acquisition
DJ-1	Protein/nucleic acid deglycase DJ-1
Dkk-4	Dickkopf-related protein 4
DSG1	Desmoglein-1
DSP	Desmoplakin
DVT	Deep vein thrombosis
EDA-A2	Ectodysplasin A2
EDTA	Ethylenediaminetetraacetic acid
EEF1D	Elongation Factor 1-Delta
EEF2	Elongation Factor 2
EFEMP1	EGF-Containing Fibulin-Like Extracellular Matrix Protein 1
ELISA	Enzyme-Linked Immunosorbent Assay
ENO1	Alpha-Enolase
EOVT	The early onset venous thrombosis
EPHB4	Ephrin type-B receptor 4
EPPK1	Epiplakin
EV	Extracellular vesicle
F13B	Coagulation Factor XIII β
Factor II	Prothrombin
FABP4	Fatty Acid Binding Protein 4
FABP6	Fatty acid binding protein 6
FARIVE	French multicentre case–control study
FARIVE	French multicenter case–control study
FETUB	Fetuin-B
FGF-6	Fibroblast growth factor 6
FGF-9	Fibroblast growth factor 9
FGG	Fibrinogen Gamma Chain
FIX	Coagulation Factor IX
FLT3LG	FMS-related tyrosine kinase 3 ligand
FXII	Coagulation factor XII
FXYD2	Sodium/potassium-transporting ATPase subunit gamma
Gal-3	Galectin-3
Gal3BP	Galectin-3 Binding Protein
GALNT3	Polypeptide N-acetylgalactosaminyltransferase 3
GAPDH	Glyceraldehyde-3-Phosphate Dehydrogenase
Gas-1	Growth arrest-specific protein 1
GC-MS	Gas Chromatography-Mass Spectrometry
GMP-VTE	Genotyping and Molecular Phenotyping in Venous Thromboembolism
GO	Gene Ontology
GPI	Glucose-6-Phosphate Isomerase
GPIb	Platelet glycoprotein Ib
GPIbA	Platelet glycoprotein Ib α chain
GPIbB	Platelet glycoprotein Ib β chain
GPIIb	Integrin α-IIb
GPIII	Integrin β-3
GPIX	Platelet glycoprotein IX
GPX3	Glutathione peroxidase 3
GSEA	Gene Set Enrichment Analysis
GSN	Gelsolin
GWAS	Genome-wide association studies
H2AFY	Core Histone Macro-H2A.1
HDL-C	High-density lipoprotein cholesterol
hEVs	Hepatocyte-Derived Extracellular Vesicles
HIVEP1	Human immunodeficiency virus type I enhancer binding protein 1
HP	Haptoglobin
HRG	Histidine-rich glycoprotein
HSPA5	78 kDa glucose-regulated protein
ICAM1	Intercellular adhesion molecule 1
ICAM2	Intercellular Adhesion Molecule 2
IC-MS	Immunocapture mass spectrometry
IDUA	α-L-iduronidase
IFN-γ	Interferon-γ
IgA1	Ig alpha-1 chain C region
IGFBP-5	Insulin-like growth factor-binding protein 5
IGHA1	Immunoglobulin heavy constant α 1
IGHV4-39	Immunoglobulin Heavy Variable 4-39
IGKV1-33	Immunoglobulin kappa variable 1-33
IGKV1D-39	Immunoglobulin Kappa Variable 1d-39
IGKV2-40	Immunoglobulin Kappa Variable 2-40
IGKV2D-40	Immunoglobulin Kappa Variable 2D-40
IGKV4-1	Immunoglobulin Kappa Variable 4-1
IGLV3-19	Immunoglobulin Lambda Variable 3-19
IL-1	Interleukin-1
IL-17C	Interleukin-17C
IL18BP	Interleukin 18 binding protein
IL6	Interleukin 6
ILK	Integrin-linked protein kinase
ITGB1	Integrin β-1
ITIH1	Inter α trypsin heavy chain H1 inhibitor
ITIH4	Inter-α-trypsin inhibitor heavy chain H4
ITIL	Inter-α-trypsin inhibitor
K1C9	Keratin type I cytoskeletal 9
KNG1	Kininogen 1
KRT6B	Keratin, Type II Cytoskeletal 6B
LAYN	Layilin
LBP	lipopolysaccharide-binding protein
LC	Liquid chromatography
LDHA	Lactate Dehydrogenase A
LDHB	Lactate Dehydrogenase B
LDL-C	Low-density lipoprotein cholesterol
LEP	Leptin
LGALSL	Galectin-Related Protein
LGALS4	Galectin 4/lectin, galactoside-binding soluble 4
LONP1	Lon protease homologue
LPS	lipopolysaccharide
LRG	leucine-rich-α-2-glycoprotein
LRG1	Leucine-rich alpha-2-glycoprotein
LRP4	Low-Density Lipoprotein Receptor-Related Protein 4
LYVE1	Lymphatic vessel endothelial hyaluronan receptor 1
MALDI	Matrix-Assisted Laser Desorption/Ionisation
MAN1A2	Mannosidase alpha class 1A member 2
MARTHA	MARseille THrombosis Association
MASP1	Mannan-binding lectin serine protease 1
MINPP1	Multiple Inositol-Polyphosphate Phosphatase 1
MMRN1	Multimerin-1
MS	Mass spectrometry
NAMLAA	N-acetylmuramoyl-L-alanine amidase
nanoESI	Nanoelectrospray ionisation
OPN	Osteopontin
ORM1	Alpha-1- Acid Glycoprotein
ORM2	Alpha-1-acid glycoprotein 2
PAI-1	Plasminogen activator inhibitor-1
PCPE	Procollagen C-endopeptidase enhancer
PCYOX1	Prenylcysteine Oxidase 1
PDGFB	Platelet-derived growth factor β
PE	Pulmonary embolisms
PEBP1	Phosphatidylethanolamine-binding protein 1
PGLYRP1	Peptidoglycan recognition protein 1
PKM	Pyruvate Kinase, Muscle Isoform
PLAT	Plasminogen activator, tissue
PLCG2	Phospholipase C Gamma 2
PLEK	Pleckstrin
PLG	Plasminogen
POF1B	Protein POF1B
POTEF	POTE ankyrin domain family member F
PROC	Protein C
PROCR	Protein C receptor, endothelial
ProZ	Vitamin K-dependent protein Z
PSMA4	Proteasome subunit alpha type-4
PTPRU	Receptor-type tyrosine-protein phosphatase U
RARRES2	retinoic acid receptor responder 2
RBP4	Retinol-binding protein 4
RETROVE	Riesgo de Enfermedad TROmboembólica Venosa
ROCK1	Rho-associated, coiled-coil-containing protein kinase 1
RPS21	40S Ribosomal Protein S21
RT-qPCR	Reverse transcription quantitative polymerase chain reaction
SAA	Serum amyloid A
SAA4	Serum Amyloid A Protein 4
SDC1	Syndecan-1
SELP	P-selectin
SERPINA1	Serpin Family A Member 1 (Alpha-1 Antitrypsin)
SERPINA10	Serpin Family A Member 10
SERPINA4	Serpin peptidase inhibitor, clade A, member 4
SERPINA5	Serpin peptidase inhibitor, clade A, member 5
SERPINE1	Serpin peptidase inhibitor, clade E, member 1
SERPINE2	Serpin Family E Member 2 (Protease Nexin-1)
SERPING1	Serpin Family G Member 1 (C1 Esterase Inhibitor)
SLC2A3	Solute carrier family 2
SPS	Synchronous Precursor Selection
ST2	ST2 protein (Interleukin-1 receptor-like 1)
STIP1	Stress-Induced Phosphoprotein 1
TAT	Thrombin-antithrombin complex
TBXAS1	Thromboxane A synthase 1
TC	Cholesterol
TF	Serotransferrin
TFPI	Tissue factor pathway inhibitor
TfR1	Transferrin receptor protein 1
THBD	Thrombomodulin
THBS2	Thrombospondin 2
THBS4	Thrombospondin 4
TLN1	Talin-1
TMT	Tandem mass tag
TNC	Tenascin C
TNFi	Tumour necrosis factor inhibitors
TNFRSF11A	Tumour necrosis factor receptor superfamily member 11A
TNFSF13B	Tumour necrosis factor ligand superfamily member 13B
TOF	Time-of-Flight
TPI1	Triosephosphate Isomerase 1
TPO	Thrombopoietin
TREML1	TREM-like transcript-1
TRY-3	Trypsin-3
TSLP	Thymic Stromal Lymphopoietin
TSP1	Thrombospondin 1
TTR	Transthyretin
TUBA4A	Tubulin α-4A chain
TUBB1	Tubulin β-1 chain
TYRO3	Transmembrane receptor tyrosine kinase
ULBP-2	UL16-binding protein 2
UMOD	Uromodulin
VCL	Vinculin
VEBIOS	Venous Thromboembolism BIOmarker Study
VEGFA	Vascular endothelial growth factor A
VSIG2	V-set and immunoglobulin domain containing 2
VTE	Venous thromboembolism
vWF	von Willebrand factor
WDR59	WD repeat-containing protein 59
WISP2	WNT1-Inducible Signalling Pathway Protein 2 (also known as CCN family member 5)
YWHAQ	Tyrosine 3-Monooxygenase/Tryptophan 5-Monooxygenase Activation Protein Theta (14-3-3 Theta)
βig-H3	Transforming growth factor-beta-induced protein ig-h3
β-NGF	β-Nerve Growth Factor

## Appendix A

### Search Terms

Search term for PubMed: ((“Venous Thromboembolism”[Mesh] OR “venous thromboembolism”[tiab] OR “venous thrombosis”[tiab] OR “deep vein thrombosis”[tiab] OR “deep venous thrombosis”[tiab] OR “pulmonary embolism”[tiab] OR “pulmonary emboli”[tiab] OR “VTE”[tiab] OR “DVT”[tiab] OR “PE”[tiab]) AND (“Proteomics”[Mesh] OR “Proteome”[Mesh] OR “proteomic*”[tw] OR “proteom*”[tw] OR “mass spectrometry”[tiab] OR “LC-MS”[tiab] OR “LC-MS/MS”[tiab] OR “tandem mass spectrometry”[tiab] OR “shotgun proteomics”[tiab] OR “protein profiling”[tiab] OR “protein expression profiling”[tiab] OR “protein signature*”[tiab] OR “quantitative proteomics”[tiab] OR “biomarker discovery”[tiab])) AND (“1995/01/01”[Date—Publication]: “2025/12/31”[Date—Publication]) AND (english[lang]) NOT (animals[Mesh] NOT humans[Mesh]).

Search term for Scopus: (TITLE-ABS-KEY (“venous thromboembolism” OR “venous thrombosis” OR “deep vein thrombosis” OR “deep venous thrombosis” OR “pulmonary embolism” OR “pulmonary emboli” OR VTE OR DVT OR PE)) AND (TITLE-ABS-KEY (proteomic* OR proteom* OR “plasma protein*” OR “serum protein*” OR “circulating protein*” OR “mass spectrometry” OR “LC-MS” OR “LC-MS/MS” OR “tandem mass spectrometry” OR “shotgun proteomics” OR “protein profiling” OR “protein expression profiling” OR “protein signature*” OR “quantitative proteomics” OR “biomarker discovery”)) AND PUBYEAR > 1994 AND PUBYEAR < 2026 AND NOT (TITLE-ABS-KEY (animal*) AND NOT TITLE-ABS-KEY (human* OR patient*)) AND (LIMIT-TO (DOCTYPE,“ar”)) AND (LIMIT-TO (LANGUAGE,“English”)).
